# Evolution of sex‐determination in dioecious plants: From active Y to X/A balance?

**DOI:** 10.1002/bies.202300111

**Published:** 2023-09-11

**Authors:** Yusuke Kazama, Taiki Kobayashi, Dmitry A. Filatov

**Affiliations:** ^1^ Graduate school of Bioscience and Biotechnology Fukui Prefectural University Eiheiji‐cho Fukui Japan; ^2^ RIKEN Nishina Center Wako Saitama Japan; ^3^ Department of Biology University of Oxford Oxford UK

**Keywords:** *CLAVATA3*, dioecious plant, gynoecium suppression, sex‐determining gene, *WUSCHEL*

## Abstract

Sex chromosomes in plants have been known for a century, but only recently have we begun to understand the mechanisms behind sex determination in dioecious plants. Here, we discuss evolution of sex determination, focusing on *Silene latifolia*, where evolution of separate sexes is consistent with the classic “two mutations” model—a loss of function male sterility mutation and a gain of function gynoecium suppression mutation, which turned an ancestral hermaphroditic population into separate males and females. Interestingly, the gynoecium suppression function in *S. latifolia* evolved via loss of function in at least two sex‐linked genes and works via gene dosage balance between sex‐linked, and autosomal genes. This system resembles X/A‐ratio‐based sex determination systems in *Drosophila* and *Rumex*, and could represent a steppingstone in the evolution of X/A‐ratio‐based sex determination from an active Y system.

## Graphical abstract

Chromosomal sex determination (SD) typically works either via active‐Y (e.g., mammals) or via X/A‐balance (e.g., *Drosophila*). The recently described SD in plant *Silene latifolia* is an active‐Y system, but it works via *WUS*‐*CLV3* gene dosage balance. We argue that this represents a steppingstone from an active‐Y to an X/A‐ratio SD.

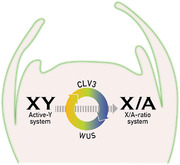

## INTRODUCTION

Sex chromosomes in flowering plants were discovered a century ago in White Campion (*Silene latifolia*), *Rumex acetosa*, *Humulus lupulus*, and *Humulus japonicus*.^[^ ^1^, ^2^, ^3^ ^]^ *S. latifolia* became a *de facto* model system for studies of sex chromosomes in plants, perhaps due to large and strongly heteromorphic sex chromosomes, making their identification easier under the microscope. Early work in this species^[^ ^4^ ^]^ has inspired the development of the classical “two mutations” (aka “two genes” or “two‐factor”) model stating that dioecy evolves via the gynodioecy pathway, in which a mutation responsible for the abortion of stamen or pollen maturation followed by another mutation causing carpel suppression.^[^ ^5^, ^6^ ^]^ This model has been favored by many plant sex chromosome researchers, probably because it naturally explains the evolution of dioecy and sex chromosomes at the same time.

The alternative “monoecy pathway” model states that dioecy evolves from monoecy via the evolution of a single sex‐determining gene.^[^ ^7^, ^8^, ^9^, ^10^ ^]^ Single‐factor sex‐determination systems were indeed found in some dioecious species, such as *Diospyros lotus* ^[^ ^11^ ^]^ and *Poplus* spp.^[^ ^12^, ^13^ ^]^ However, it is worth noting that even in single‐factor sex‐determining systems more than one mutation may be necessary for the emergence of dioecy; for example, the evolution of both sex‐determining genes, *MeGI* and *OGI* would be required for dioecy in *D. lotus*.

Early genetic work in *S. latifolia* has revealed the presence of two sex‐determining genes on the Y‐chromosome, the gynoecium suppressing factor (*GSF*) and stamen promoting factor (*SPF*).^[^ ^4^ ^]^ The deletion of *GSF* or *SPF* sex‐determining genes leads to the development of hermaphroditic or asexual flowers, respectively,^[^ ^14^, ^15^ ^]^ which is consistent with the “two‐factor” model for sex determination and sex chromosome evolution.^[^ ^5^, ^6^ ^]^ Similarly, in asparagus and kiwifruit sex determination is controlled by two genes. In kiwifruit, *Shy Girl* (*SyGl*) and *Friendly Boy* (*FrBy*) act as the pistil‐suppressing and stamen‐promoting genes, respectively.^[^ ^16^, ^17^ ^]^ In asparagus, *SUPPRESSOR OF FEMALE FUNCTION* (*SOFF*) and *DEFECTIVE IN TAPETAL DEVELOPMENT AND FUNCTION1* (*TDF1*) were identified as the pistil‐suppressing and stamen‐promoting genes, respectively.^[^ ^18^, ^19^, ^20^ ^]^ However, the sex‐determining region on the Y‐chromosome is hemizygous so that the X chromosome has no gametologs of these sex determining genes,^[^ ^18^ ^]^ making it difficult to reconstruct the origin of sex‐determining genes during sex chromosome evolution. Identification of sex‐determining genes in *S. latifolia* ^[^ ^21^ ^]^ offers an opportunity to reveal how the sex‐determination and large heteromorphic sex chromosomes evolve from scratch.

## EVOLUTION OF GYNOECIUM SUPPRESSION IN *S. LATIFOLIA* VIA TWO GENE LOSSES

**FIGURE 1:**
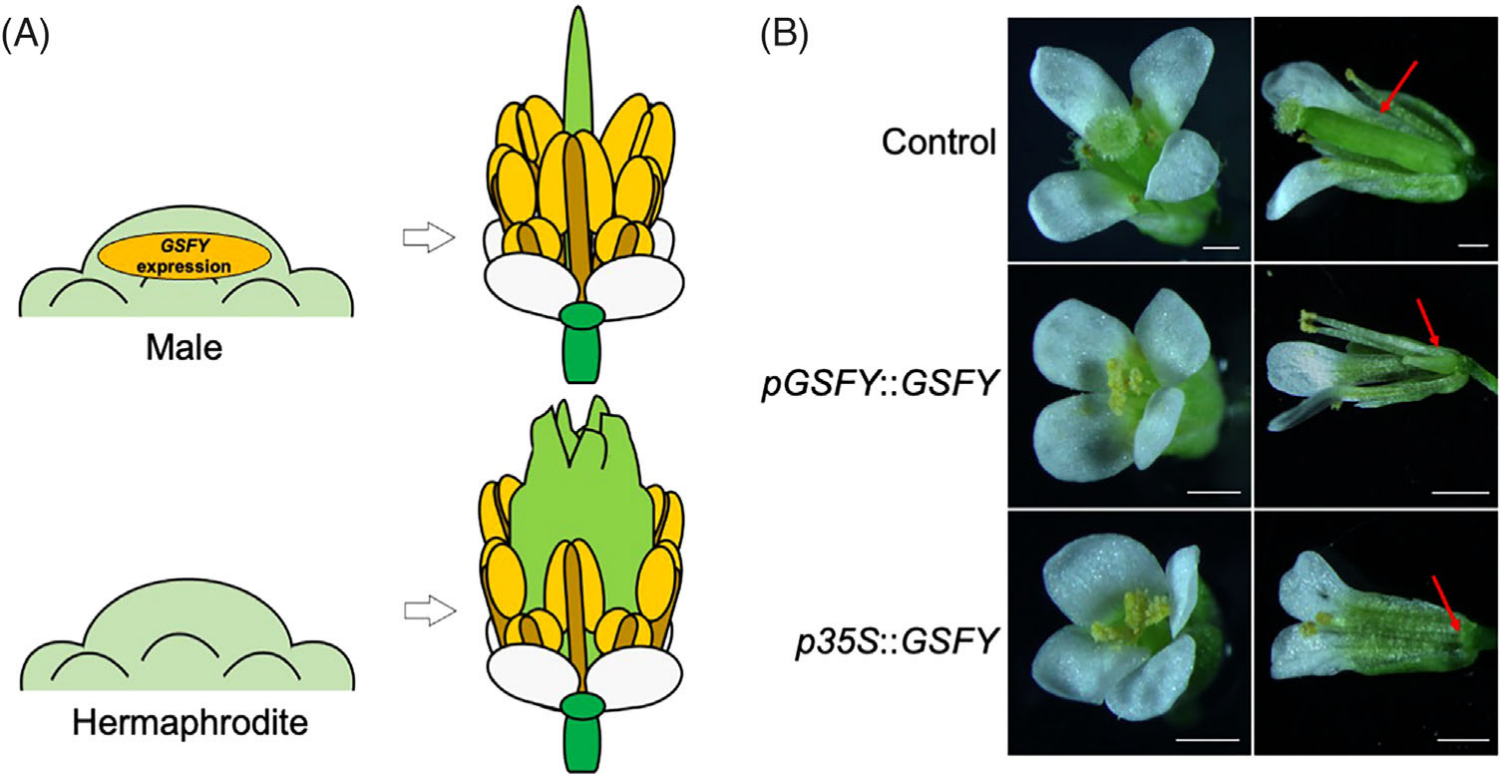
Function of *GSFY* gene in gynoecium suppression. (A) Expression of *GSFY* at the early stages of flower development causes the suppression of the gynoecium. (B) Phenotypes of *Arabidopsis* transformants in which *GSFY* was introduced under the control of the native promoter and the CaMV35S promoter. Bars = 1 mm.

Detailed deletion mapping of the *S. latifolia* Y‐chromosome,^[^ ^22^, ^23^ ^]^ combined with genomic sequencing of the hermaphroditic mutants and functional molecular genetic analyses, revealed the most likely candidate for the *GSF* gene.^[^ ^21^ ^]^ This gene is homologous to the *Arabidopsis CLAVATA3* (*CLV3*) and it has functional Y‐linked (*GSFY*) and dysfunctional X‐linked (*GSFX*) gametologs that diverged around the time when *S. latifolia* sex chromosomes originated.^[^ ^21^ ^]^ *CLV3* gene regulates the size of the shoot apical meristems (SAMs) and flower bud primordia in *Arabidopsis*.^[^ ^24^ ^]^ The *clv‐3* mutants in *Arabidopsis* often show enlargement of carpels, while overexpression of *CLV3* leads to pistil‐less flowers.^[^ ^24^ ^]^ These phenotypes are similar to those of female and male flowers of *S. latifolia*, respectively (Figure 1A), suggesting that this gene is a likely candidate for *GSF*. This was also confirmed by transgenic analyses (Figure 1B) and bioassays in which *A. thaliana* and *S. latifolia* were treated with synthetic peptides derived from GSFX/Y, CLV3, and the mutant allele of CLV3 (CLV3m).^[^ ^21^ ^]^

Based on the available evidence, the *GSF* function in *S. latifolia* likely works via the *WUSCHEL‐CLAVATA* (*WUS*‐*CLV*) feedback loop^[^ ^21^ ^]^ where the *CLV3* mRNA production is activated by WUS protein, while the expression of *WUS* is repressed by *CLV3*. In *Arabidopsis*, higher concentration of *WUS* represses the transcription of CLV3.^[^ ^25^, ^26^, ^27^ ^]^ The *S. latifolia* gene encoding a counterpart of *CLV3* in this feedback loop, *SlWUS1*, is linked to the X chromosomes, but absent from the Y chromosome.^[^ ^28^ ^]^ In *Arabidopsis*, *wus* mutants show the loss of pistil^[^ ^29^ ^]^ and in *S. latifolia* the X‐linked *SlWUS1* appears to control the size of gynoecium. This was supported by the observation of smaller gynoecium size and the smaller numbers of seeds in plants with fewer copies of *SlWUS1*.^[^ ^30^, ^31^ ^]^ A possible effect of the copy number of *SlWUS1* on gynoecium size can also be found in the classic analyses of colchicine‐treated polyploid plants.^[^ ^32^ ^]^ As the ratio of the X chromosomes to the Y chromosome increased, hermaphroditic flowers were produced more frequently and in the case of XXXXY plant, almost all flowers were hermaphrodites. It is interesting that *SlWUS1* is not dosage compensated (i.e., not upregulated in males)^[^ ^28^ ^]^ despite the location in the oldest stratum 1^[^ ^33^ ^]^ where we may expect dosage compensation to be prevalent, particularly given the recent models predicting that dosage compensation is responsible for evolution of X‐Y recombination repression.^[^ ^34^, ^35^ ^]^ This likely reflects the dosage sensitivity of *SlWUS1* that should not be altered by evolving dosage compensation system. Taken together, the *S. latifolia* X chromosome is apparently involved in sex determination with X/Y dosage balance mechanism, that likely operates via the *WUS*/*CLV* feedback loop.^[^ ^21^ ^]^

According to the canonical two‐factor model, the transition from ancestral hermaphroditism to dioecy via gynodioecy involves a recessive loss‐of‐function male sterility mutation on the proto‐X and a dominant gain‐of‐function female sterility mutation on the proto‐Y‐chromosome.^[^ ^5^ ^]^ Thus, the finding of an X‐linked loss‐of‐function mutation in *GSFX* ^[^ ^21^ ^]^ may seem surprising. However, the presence of functional *GSFY* on the Y‐chromosome effectively plays the role of a dominant gain‐of‐function Y‐linked female sterility gene that is predicted by the canonical two‐factor model. This scenario suggests that not just two but at least three mutations, one for maleness (not yet identified) and two for femaleness (dysfunctionalizing *GSFX* on the X and *SlWUS1* on the Y) might have been involved in the transition from hermaphroditism to full dioecy in *S. latifolia*. The evolution of female suppression via more than one mutation is not surprising, given the loss of female function is costly in terms of fitness and must be offset by at least a two‐fold gain in reproductive success via male function.^[^ ^36^ ^]^ Thus, splitting this costly change into several steps, each of which reduces female function, may be a more realistic way to complete the shift from gynodioecy to dioecy.

## DOES THE *WUS*/*CLV3* BALANCE REPRESENT A STEPPINGSTONE IN EVOLUTION OF X/A‐RATIO‐BASED SEX DETERMINATION?

**FIGURE 2:**
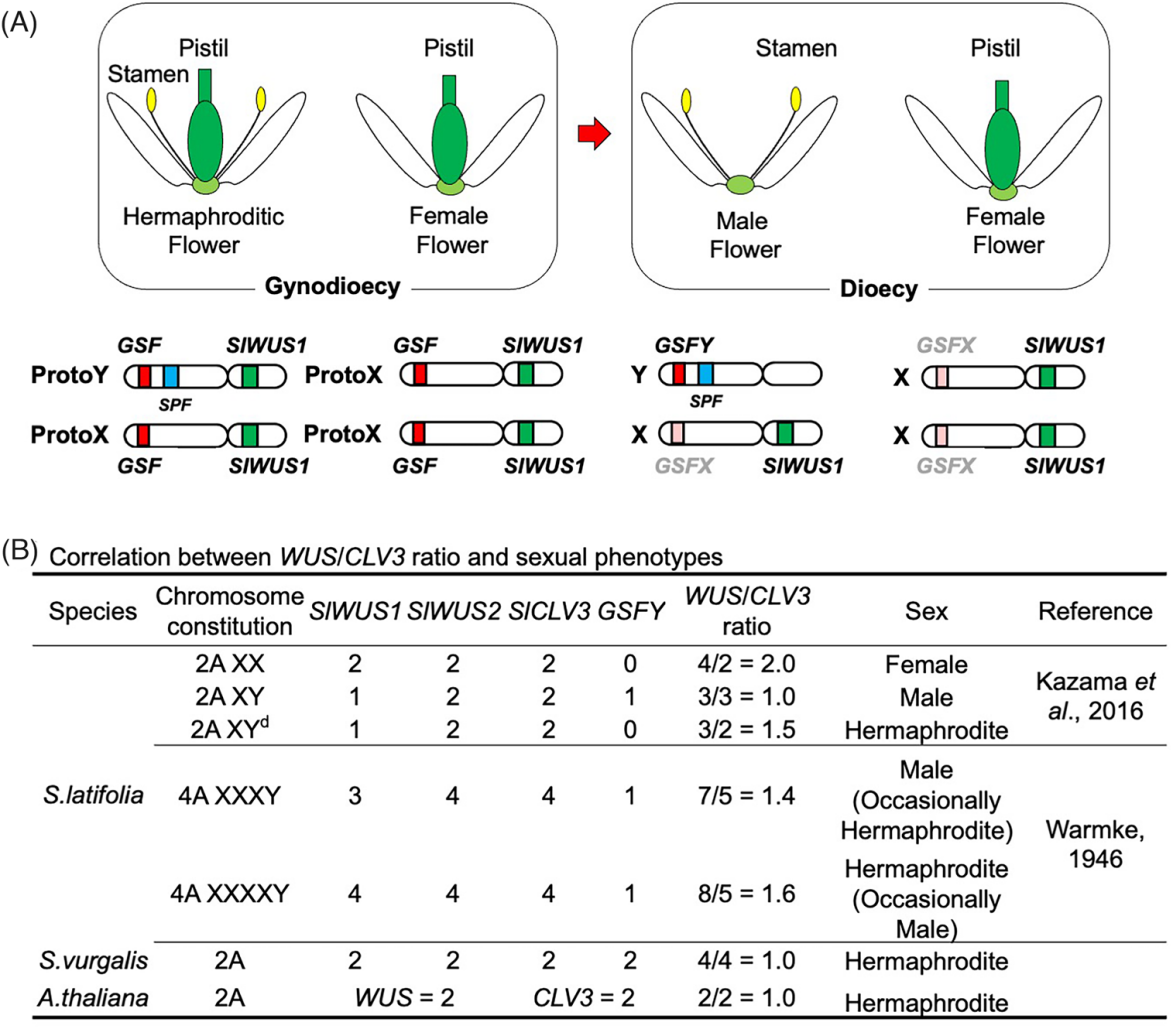
Gynoecium suppression mechanism in *S. latifolia*. (A) Ideogram for the evolution of gynoecium suppression in *S. latifolia*. The protoX/Y chromosomes would have had ancestral *GSF* and *SlWUS1* genes. *GSF* was dysfunctionalized on the X, leaving the active *GSFY* gene on the Y chromosome, while *SlWUS1* disappeared from the Y chromosome, but remains functional on the X‐chromosome. (B) Correlation between *WUS*/*CLV3* ratio and sexual phenotypes. The *WUS*/*CLV3* ratio well explains sexual phenotypes of *S. latifolia*; 2 is female, 1.5 is hermaphrodite, and 1 is male. This ratio threshold has likely evolved upwards from the original hermaphroditic state seen in *S. vulgaris* and *A. thaliana*.

If gynoecium suppression in *S. latifolia* is controlled via the WUS/CLV feedback loop,^[^ ^21^ ^]^ we would expect the ratio of *WUS* and *CLV3* copy numbers in a plant to predict the size of its gynoecium. To test this expectation, we also have to take into account the presence of expressed autosomal paralogs (Kazama et al.,^[^ ^28^ ^]^ and unpublished) of these genes in *S. latifolia* genome—*SlWUS2* and *SlCLV3*. Consistent with the above expectation, the copy number difference of *WUS* and *CLV3* genes between males and females explains the sex of *S. latifolia* flowers well (Figure 2B). In male, female, and hermaphroditic mutant plants, the *WUS*/*CLV3* ratios are 1, 2, and 1.5, respectively. These ratios are consistent with chromosome numbers in the synthetic polyploid *S. latifolia* plants.^[^ ^32^ ^]^ The XXXY plants (with *WUS*/*CLV3* ratio = 1.4) are males with occasional hermaphroditic flowers, while the XXXXY plants (*WUS*/*CLV3* = 1.6) are hermaphrodites with occasional male flowers (Figure 2B). Therefore, the *WUS*/*CLV3* ratio appears to explain the mechanism of gynoecium suppressing functions in *S. latifolia*. Furthermore, it was reported that treatment of males with hypomethylating chemical 5‐azacytidine results in occasional development of hermaphroditic flowers, either due to suppression of a Y‐linked female suppressor gene, or due to activation of autosomal female‐promoting gene,^[^ ^37^ ^]^ which can be explained by the gene balance model in the WUS‐CLV feedback loop controlling the development of female organs with X‐ and autosomal‐linked *WUS* genes in *S. latifolia* flowers (Figure 2B).

In the case of hermaphroditic species, such as *S. vulgaris* and *A. thaliana*, the *WUS*/*CLV3* ratio is 1, indicating that the threshold for gynoecium suppression in the *WUS*/*CLV3* ratio in the dioecious *S. latifolia* has evolved during dioecy evolution in *S. latifolia* ancestor. It is interesting to speculate that this system could evolve further to eventually become an X/A‐ratio system when the Y chromosome degenerates and the Y‐linked *GSFY* gene is lost or moved to an autosome. Such X/A‐ratio sex determination is observed in *R. acetosa* ^[^ ^38^, ^39^ ^]^ and *Drosophila melanogaster*.^[^ ^40^, ^41^ ^]^ Interestingly, genus *Rumex* includes dioecious species with the X/A‐ratio as well as with the active‐Y sex‐determining mechanisms,^[^ ^42^ ^]^ which is consistent with the suggestion of Westergaard that the X/A‐ratio systems evolve secondarily from the active‐Y system.^[^ ^5^ ^]^

The molecular bases of X/A‐ratio‐based sex determination in *D. melanogaster* are well‐studied.^[^ ^43^ ^]^ Sex in *D. melanogaster* is controlled by the ratio of the products of X‐linked “numerator” (sisA, sisB, sisC, and runt) and autosomal “denominator” (deadpan, Dpn) genes in early development.^[^ ^43^ ^]^ The products of the numerator genes, collectively called X‐linked signal elements (XSE), are transcription factors that activate expression of the key sex‐determining gene *sexlethal* (*Sxl*), while the denominator Dpn acts as a repressor of *Slx*. The ratio of X‐linked nominators and autosomal denominator proteins is sufficient to activate *Sxl* in females (XX: AA), but not in males (X:AA).^[^ ^43^ ^]^ Once activated, the expression of *Sxl* is self‐maintained throughout the female body and it controls the splicing of downstream genes in sex determination pathway. The functional SXL in females plays a role in splicing a splicing regulator *transformer* (*tra*) into a functional isoform, whereas lack of the functional SXL in males leads to nonfunctional splicing of *tra*,^[^ ^44^, ^45^ ^]^ resulting in male specific splicing of the well‐conserved sex‐determination related gene, *doublesex* (*dsx*).^[^ ^46^ ^]^ The male‐specific *dsx* promotes the development of male morphology. This X:A ratio system is thought to evolve from the ancestral XY system via sex chromosome turnover with recruitment of *Slx* to the X chromosomes.^[^ ^47^ ^]^

Similar to sex‐determination in *D. melanogaster*, the female‐suppressing function in *S. latifolia* is controlled by gene dosage balance, with the X‐linked positive regulator (*SlWUS1*), but unlike *D. melanogaster*, the negative regulator (*GSFY*) is Y‐linked, resulting in an active‐Y rather than X/A‐ratio system. However, ongoing Y‐degeneration^[^ ^48^, ^49^ ^]^ may lead to eventual *GSFY* loss and evolution of X/A‐ratio system controlled by the number of X‐linked *WUS* genes. Interestingly, *WUS* is also involved in the sex expression pathway in kiwifruits,^[^ ^16^ ^]^ and *Cucumis*,^[^ ^50^ ^]^ implying that this mechanism possibly plays a role in gynoecium suppression of many plant groups with independently evolved dioecy and sex chromosomes. Further analyses of sub‐functionalization of *GSFY* or *SlWUS1* and the effect of their copy numbers will allow one to test whether *WUS*/*CLV3* ratio control the gynoecium suppression.

## CONCLUSIONS

The gene dosage balance‐based gynoecium suppression in *S. latifolia* may be regarded as a steppingstone in the evolution of an X/A balance sex determination system, such as found in *Drosophila* and *Rumex*. The tendency of Y‐linked genes to undergo genetic degeneration may mean that such shifts from an active‐Y to X/A balance‐based sex determination is a general phenomenon. The mechanism of sex determination in *S. latifolia* provides an illustration of how an ancestral active‐Y sex determination could be modified to turn it into an X/A balance‐based system. The eventual loss of *GSFY* due progressing Y‐degeneration in *S. latifolia* may be compensated by the modulation of the *WUS* gene expression level, giving rise to the development of the X/A‐ratio system. Further investigations into the molecular bases of this system in *S. latifolia* will provide valuable insights into the dynamic process of sex‐chromosome evolution and enhance our understanding of sex‐determination mechanisms in plants generally.

## AUTHOR CONTRIBUTIONS

All authors contributed to the writing of this manuscript.

## CONFLICT OF INTEREST STATEMENT

The authors declare no conflicts of interest.

## Data Availability

Data sharing not applicable as no new data generated.
